# Intention to receive Latent Tuberculosis Infection (LTBI) treatment and its associated factors among healthcare workers in a Malaysian teaching university hospital

**DOI:** 10.1371/journal.pone.0307199

**Published:** 2024-07-18

**Authors:** Wan Muhammad Zainol Zakaria, Zawiah Mansor

**Affiliations:** Department of Community Health, Faculty of Medicine and Health Sciences, Universiti Putra Malaysia, Selangor, Malaysia; Zagazig University Faculty of Human Medicine, EGYPT

## Abstract

The acceptability of latent tuberculosis infection (LTBI) therapy remains low among healthcare workers (HCWs). Up to 10% of LTBI cases can reactivate into active tuberculosis, posing risks to HCWs and patients. Understanding HCWs’ intention to undergo LTBI treatment is crucial for designing effective management policies, especially where no LTBI policy exists. This cross-sectional study investigated the intention to receive LTBI therapy and its associated factors among HCWs in a Malaysian teaching hospital. The study was conducted from 5^th^ to 30^th^ May 2023, in a hospital without an LTBI screening program. Stratified random sampling was used to select HCWs, excluding those undergoing TB or LTBI therapy. Respondents completed a questionnaire measuring intention to receive LTBI treatment, LTBI knowledge, attitude, perceived norm, and perceived behavioral control. Of the 256 respondents, the majority were female (63.7%), under 35 years old (64.45%), had no comorbidities (82.0%), and worked in clinical settings (70.3%). However, 60.5% of respondents had low LTBI knowledge and 60.5% held unfavorable attitudes toward LTBI treatment. Despite this, 53.5% of respondents intended to undergo LTBI therapy if diagnosed. Factors positively associated with this intention included being female [aOR: 2.033, 95% CI: 1.080–3.823], having high LTBI knowledge [aOR 1.926, 95% CI: 1.093–3.397], had favorable attitude [aOR 3.771, 95% CI: 1.759–8.084], and strongly perceiving social norms supportive of LTBI treatment [aOR 4.593, 95% CI: 2.104–10.023]. These findings emphasize the need for an LTBI management policy in the teaching hospital. To boost HCWs’ intention and acceptance of LTBI treatment, a focused program improving knowledge, attitude, and perception of social norms could be introduced.

## Introduction

Latent Tuberculosis Infection (LTBI) refers to a persistent immune reaction that derives from dormant Tuberculosis (TB) infection by *Mycobacterium Tuberculosis* without showing any active TB symptom or sign [1, 2]. Following TB bacterial exposure, one may develop active TB (30.0%), LTBI (90.0–95.0%), and total eradication (70.0%) of TB bacteria from the body. Although LTBI is a non-infectious disease when compared to active TB, the significance of public health resides in the potential reactivation into active TB. As depicted in the World Health Organisation (WHO) LTBI guideline, 5–10% of healthy adults with LTBI will develop reactivation of LTBI at some point in their lifetime, particularly within the first five years of LTBI [2]. Immunity status is significantly correlated with TB reactivation from LTBI, along with immunocompromised conditions such as diabetes mellitus, HIV, renal transplant, and cancer that escalate LTBI reactivation [2, 3]. Similar to the global concern, TB remains a major public health concern and priority in Malaysia. Current issues, such as multiresistant drug TB and LTBI, which can contribute to Malaysia’s increasing TB burden, must be investigated in order to mitigate their impact [4–6].

Interferon Gamma Release Assay (IGRA) and Tuberculin Skin Test (TST) are immune-based reaction tests for LTBI diagnosis [3, 7]. The IGRA test (e.g., QuantiFERON-TB Gold Plus (QFT- Plus)) refers to a modern *in vitro* blood test applied to detect gamma interferon (IFN-γ), which is released by activated T lymphocytes in response to TB antigens [8, 9]. Besides identifying CD8+ and CD4+ responses to TB infection, QFT-Plus has higher specificity and sensitivity than TST [10–13]. The absence of LTBI gold standard has caused its worldwide uncertain prevalence. The WHO predicts that one-quarter of the global population suffers from LTBI. Healthcare workers (HCWs) are exposed to the risk of contracting LTBI 9.3% higher than the public, especially in high TB burden areas [2, 14]. The risk of HCWs contracting LTBI is in proportion to the rate of TB incidences in a country [15]. In low- and middle-income countries (LMIC), ~ 30.0% [95% CI = 19.0–41.0] of HCWs encountered LTBI, while 56.0% [95% CI = 39.0–73.0] was recorded in countries with high TB burden [15]. As an LMIC with intermediate TB burden, Malaysia recorded ~ 10.6% [95% CI = 8.6% - 12.6%] prevalence of LTBI amidst HCWs upon being tested with IGRA [15, 16].

Treatment of LTBI is vital to eradicate TB, as depicted in *WHO End TB Strategy 2035* to achieve 90% coverage of TB preventive therapy. However, LTBI treatment programs are mostly executed in high-income countries with low TB burden [2, 17]. To bridge the time gap in policy formulation, the initial edition of LTBI management published by the WHO in 2015 was substituted with an updated version of a consolidated LTBI management guideline policy in 2018 [2]. The LTBI program is a novel policy across developing countries. Despite the establishment of the National Tuberculosis Control Program in 1961 in Malaysia, detailed LTBI treatment guidelines were only issued in 2021 due to a directive order from the Director of Disease Control Division in the Malaysian Ministry of Health (MOH)–evidencing a wide time gap in policy formulation [7, 18]. Concerns about the efficacy of policy deployment in addressing LTBI in Malaysia have sparked over such delay. The directive order issued in 2021 outlined the criteria for LTBI screening and treatment by using IGRA, particularly for persons who had close contact with smear-positive TB patients, those in TB clusters, as well as HCWs willing to undergo screening and treatment. The treatment regimens of anti-TB medication include Isoniazid, Rifampicin, and Rifapentine for 3–12 months [19]. The acceptance rate of LTBI treatment among HCWs in low TB burden countries was rather low at 28% [19]. In 2021, a pilot study disclosed a similar low LTBI treatment acceptability rate in the Malaysian population [7]. Evidently, the actual behaviour of LTBI treatment acceptance has been rather low among HCWs and the public. In Malaysia, the IGRA test is mostly performed for the public but limited to HCWs serving the MOH. In MOH facilities, LTBI screening for HCWs is routinely performed at the pre-employment, pre-placement, and pre-retirement stages. Despite the recent efforts to establish LTBI programs in Malaysia, a wide gap is noted in policy coverage for HCWs working outside of the MOH, including those working in public university hospitals and private healthcare centres. The existing standards and screening practises practiced in Malaysia may put a sizable fraction of HCWs at risk of contracting LTBI. With limited screening and treatment policies in place for HCWs operating outside of government health institutions, little is known about their intention or acceptance of LTBI treatment. Nonetheless, it is a critical input to policymakers in ensuring that the LTBI program is executed fairly for all HCW in Malaysia.

Determining the acceptance of LTBI treatment is paramount in ensuring the success of TB eradication strategy. Most studies focused on population acceptance of LTBI treatment following screening investigation. The intention to receive treatment is important to predict the likelihood of one to undergo LTBI treatment prior to screening, thus facilitating to devise an effective TB eradication strategy. Intention to receive LTBI treatment refers to one’s plan to undergo the necessary treatment if found positive for LTBI. However, studies pertaining to the intention to receive LTBI treatment are in scarcity when compared to investigations that delved into the actual behaviour of LTBI treatment acceptance upon diagnosis [7, 20, 21]. In view of HCW, this health seeking intention and behaviour need special attention as the disease may become active TB at any point in time and may affect their health and patients under their care. The Integrated Behaviour Model (IBM) is a theoretical framework that depicts intent to carry out certain behaviour as the most significant determinant, which is impacted by perceived behavioural control, attitude, and perceived norm [22, 23]–reflecting constructs from Theory of Planned Behaviour (TPB) and Theory of Reasoned Action (TRA). By investigating behavioural intention using IBM, a deeper understanding of the modifiable factors may be identified to devise effective interventions that increase the acceptance rate of LTBI treatment [22, 23]. The literature depicts that the intention among HCWs to receive LTBI treatment may be influenced by occupational and sociodemographic factors. For instance, a study found that male HCWs were more likely to undergo LTBI treatment than their female counterpart [21]. This is ascribable to the fact that male HCWs have a higher likelihood of developing active TB because they are the primary breadwinners in developing countries and establish more social contacts when compared to female HCWs [24–26]. Besides, the prevalence of smoking habit among males increases lung infection risks [27–29]. These high-risk factors lead to the high rate of LTBI treatment acceptance among male HCWs. Occupational factors also can significantly influence the acceptance of LTBI treatment among HCWs. Doctors with doctorate-level qualifications and scientists displayed the lowest level of LTBI treatment acceptance, mainly due to their demanding schedules and knowledge about the potential side effects [21, 30]. The absence of clinical symptoms and healthy appearance in LTBI patients can cause HCWs to underestimate the lurking infection risks. In addition, they may perceive LTBI treatment as ineffective due to the potential for continued exposure to TB patients at healthcare facilities [30].

This study investigated the intention to receive LTBI treatment among HCWs in a teaching hospital located in Malaysia by placing a focus on factors based on the IBM theoretical framework. This study measured intention to receive LTBI treatment, as there was no compulsory policy on LTBI treatment practice in all types of healthcare facilities in Malaysia. [7]. Given that the reported outcomes shed light on LTBI treatment intention, the relevant stakeholders and policymakers may use the findings to develop targeted policies that contribute to the overall goal of eradicating TB.

## Materials and method

### Study location

A teaching university hospital located in Malaysia that focused on executing medical- based research work, training healthcare professionals, as well as offering cutting-edge diagnostic and treatment modalities was selected as the study location [31, 32]. The selected hospital was located in a highly-populated district with high TB burden as a result of high industrial activities in that area and a massive number of immigrant workers residing there [33, 34]. The Public Health Unit of the hospital recorded an increment of up to 40 TB cases in 2022 when compared to 20 cases in 2021 [35]. In Malaysia, based on directives from the Ministry of Health, Malaysia (MOH) applicable to government health facilities, the responsibility for the LTBI screening program and management lies with the Communicable Disease Sector, MOH. Screening is performed using IGRA for all HCWs. However, in private health facilities or university hospitals, LTBI screening and management are initiatives of the facilities themselves and are not compulsory [7, 18]. At the time this study was conducted, the LTBI policy was not completely implemented in the study location. Hence, the screening and treatment of LTBI was not performed routinely for HCW and the formal number of LTBI cases among the HCWs was not available.

### Study design, study population and sampling method

This cross-sectional study was conducted from 5th May 2023 to 30th May 2023 among HCWs in the teaching university hospital. In this study, the sample size was determined by computing the two-proportion formula for hypotheses testing prescribed by Lwanga and Lemeshow (1990) [36]. The sample size with the power of study set at 80%, 20% non- response rate from online questionnaires, as well as 10% non-eligibility after adhering to inclusion and exclusion criteria, was 256 respondents. The stratified random sampling method was used and the study population was stratified based on the occupational groups strata namely clinical (professionals and allied health) and non-clinical (supporting and administrative). The respondents for each strata were then selected using simple random sampling method and proportionate to the size of each strata.

### Study instrument

A self-administered questionnaire was emailed to all respondents by using Google Forms link so that they could complete the survey at their convenience without disrupting their work. The email addresses of the respondents were retrieved from the hospital website directory. To encourage the participation of the respondents in completing the survey, the head of departments in the hospital were consulted without being involved directly with data collection process. The questionnaire comprised of two main sections that incorporated 35 items to explore the intention of HCWs to receive LTBI treatment and the influential factors. The questionnaire items were adapted from prior studies with some modifications to accommodate the requirements for measuring the independent and dependent variables in this study because of the limited questionnaires that have been used to examine intention towards LTBI treatment [37–39].

Section A of the survey captured the characteristics of the respondents based on five aspects: (1) sociodemographic data (age, gender, highest level of education, & annual household income), (2) occupational history (job categories & workstation), (3) risk behaviour (smoking & alcohol consumption statuses), (4) comorbidity, and (5) TB close contact history. Section B of the survey consists of IBM constructs, including intention among HCWs to receive LTBI treatment, LTBI knowledge, perceived behavioural control, attitude, and perceived norm. Items for LTBI knowledge retrieved from Al Faleh et al., (2022) in the Arab Saudi context were validated and examined with Cronbach Alpha = 0.80 [38]. The construct of LTBI knowledge has six items, whereby one point is awarded for the selection of a correct response but no point is deducted for selecting an incorrect response. The total score was converted into percentage by dividing the sum by the maximum score and multiplying it with 100. The percentage was categorised based on Bloom’s cut-off points: values < 80% reflect low LTBI knowledge and values ≥ 80% denote high LTBI knowledge [40].

The survey items that covered IBM constructs were obtained from a past study that also embedded perceived behavioural control, attitude, and perceived norm [37]. The scale of direct measures was adopted from a questionnaire that was already validated in the past [22]. The eight items used to assess attitude are explained as follows: The initial four items are seven-point semantic differential adjective pairs (good-bad, important- unimportant, harmful-beneficial, & favourable-unfavourable) that apply reverse-coding, with a higher score denoting a more favourable attitude. The remaining four items deploy the five-point Likert scale that ranges from ‘strongly disagree’ to ‘strongly agree’ to assess the attitude of the respondents towards LTBI treatment [39]. The total score for the eight items was converted into percentage. Bloom’s cut-off point was used to classify the attitude as either favourable or unfavourable: scores ≥ 80% are favourable while scores of 79 and less are unfavourable [40]. The Cronbach alpha for attitude adapted from a past study was 0.89 [37]

The construct of perceived norm has three items and they were retrieved from a study that displayed a Cronbach alpha value of 0.73 [37]. The items use the seven-point Likert scale ranging from 1 (strongly disagree) to 7 (strongly agree). The composite score of the three items was obtained by dividing the sum of the total answer scale by three (construct item number). The mean composite score of perceived norm in this study; 3.510±0.643, was the cut-off point used to categorise strongly and weakly perceived norm: scores of 3.510 and above reflect strongly perceived norm while scores of 3.509 and below signify weakly perceived norm.

The three items for perceived behavioural control were measured by using the seven- point Likert scale that ranged from 1 (strongly disagree) to 7 (strongly agree). The Cronbach alpha score was 0.70 [37]. The composite score of the items was calculated by summing the total scale answer and dividing by three (total items of the construct). The mean composite score of 5.110±1.184 was the cut-off point used to group strongly and weakly perceived behavioural control; scores of 5.110 and above signify strong perception while scores of 5.109 and below denote weak perception.

In this study, intention to receive LTBI treatment was examined by deploying four items obtained from a past study (Cronbach alpha = 0.82) [37]. A seven-point scale that ranged from 1 (definitely don’t) to 7 (definitely do) was used by the respondents to rate their opinions or experiences. The composite score of the four-item construct was computed by dividing the sum of the answers by four (construct item number). The mean composite score of 5.760±1.134 was applied to categorise strong and weak intention: scores equal to and above 5.760 signify strong intention while scores of 5.759 and below denote weak intention. Items with negative responses were reverse-coded to assess the responses in a similar direction.

The original text of the adapted questionnaire was written in the English language and was translated to Bahasa Melayu by using the forward-backward translation approach. Three public health medicine specialists knowledgeable in IBM were selected to assess the content validity of the questionnaire. Most of the items scored 1.0 and 0.87 for Content Validity Ratio (CVR) and Content Validity Index (CVI), respectively. A pilot study that involved a similar population was carried out to ensure the validity and reliability of the questionnaire within the local context. The reliability of the questionnaire from the pilot study showed acceptable Cronbach Alpha values of 0.7–0.8.

### Data analysis

The data gathered from the respondents were sorted, coded, keyed in, and cleaned using IBM SPSS v28 for Windows. While descriptive analysis was conducted on all variables, the continuous variables were assessed by using the normality test. Next, the relationships between the intention among HCWs to receive LTBI treatment and each independent variable were analysed using bivariate analysis (i.e., Fisher’s Exact & Chi- Square tests). The predictors of intention to receive LTBI treatment were determined based on multivariate analysis via multiple logistic regression analysis. The results are presented in the form of adjusted odd ratios (aOR) with a 95% confidence interval (CI). Variables with *p*-value = 0.25 in univariate analysis were embedded into the multivariate model. After that, the variables were examined for multicollinearity. The Backward Wald approach was used in the multivariate analysis to yield a parsimonious model that fits well with the data. Since only relevant variables were incorporated into the model to describe the result, both interpretability and predictability of the model were preserved. The statistical analysis had a 0.05 significance level.

### Ethical approval

This study was registered under the National Medical Research Register (NMRR ID-23-00919-XLH). Ethical approval for this study was granted by the Ethics Committee for Research Involving Human Subjects in Universiti Putra Malaysia (JKEUPM-2023-197). In addition, permission was obtained from the Director of the selected hospital and all respondents provided informed written consent prior to the survey.

## Results

As all 256 respondents participated in the survey, this study recorded a 100% response rate. Table 1 tabulates the characteristics of the respondents. A majority of the respondents were 35 years and below (64.45%) and their median age was 33 years (n = 6). Most of the respondents were female (63.7%), had tertiary-level education (90.0%), and experienced no comorbidity (82.0%). Only a few of the respondents were smokers (10.20%) and consumed alcohol (1.20%). The median value of annual household income in Ringgit Malaysia (RM) was 48,000 with an interquartile range (IQR) of 39,600. As for the occupational groups, most respondents derived from the professional group (57.0%), followed by allied health (15.2%), supportive (14.1%), and administrative (13.7%) groups. The figures were proportionate to the enrolment size. In total, 62.9% of the respondents worked in high-risk areas. Tapping into the IBM constructs, most of the respondents had low LTBI knowledge (60.5%), along with an unfavourable attitude (60.5%), weakly perceived norm (62.9%), and weakly perceived behavioural control (52.3%) towards LTBI treatment. Notably, a majority of the respondents (53.5%) displayed positive intention to receive LTBI treatment.

**Table 1:** Sociodemographic, comorbidity, risk behaviour, occupational factors, and IBM constructs among HCWs.

Characteristics	Total (n, %)
**Total**	256
Sociodemographic	
**Age (years),** median [IQR]	33 [6]
**Age category (years)**	
< 35 years old	165 (64.5)
≥ 35 years old	91 (35.5)
**Gender**	
Female	163 (63.7)
Male	93 (36.3)
**Education level**	
Secondary level	18 (7.0)
Tertiary level	238 (93.0)
**Annual household income (RM), median [IQR]**	48,000 [39,000]
**Annual household income category (RM)** *	
< 58,200	161 (62.9)
≥ 58,200	95 (37.1)
**Comorbidity**	
Yes	46 (18.0)
No	210 (82.0)
**Smoking status** **	
Yes	26 (10.2)
No	230 (89.8)
**Alcohol consumption**	
Yes	3 (1.2)
No	253 (98.8)
**Occupational groups**	
Clinical	185(72.3)
Non clinical	71 (27.7)
**Workstation** ** *	
High-risk	161 (62.9)
Non-high-risk	95 (37.1)
**History of TB Close contact** ****	
Yes	10 (3.9)
No	246 (96.1)
**Level of LTBI knowledge**	
High	101 (39.5)
Low	155 (60.5)
**Attitude towards LTBI treatment**	
Favourable	101 (39.5)
Unfavourable	155 (60.5)
**Perceived norm towards LTBI treatment**	
Strong	95 (37.1)
Weak	161 (62.9)
**Perceived Behavioural Control towards LTBI treatment**	
Strong	122 (47.7)
Weak	134 (52.3)
**Intention to receive LTBI treatment**	
Strong	137 (53.5)
Weak	119(46.5)

*The annual household income is classified based on B40 and non-B40 groups [41].

** Smoking refers to one who smokes at least 10 cigarettes daily or intermittently [42, 43].

*** High-risk workstation incorporates internal medicine wards, infection control units, outpatient clinics, clinical laboratory, isolation wards, intensive care units, and emergency department [44].

**** One who shares a similar enclosed living space with the index case of TB for one or more nights or for a frequent or extended amount of time over the three months prior to the present treatment episode [45].

Associations among sociodemographic factors, risk behaviour, occupational factors, TB close contact history, comorbidity, and intention of HCWs to receive LTBI treatment are presented in Table 2. The table also demonstrates the relationships of IBM constructs (i.e., LTBI knowledge, perceived behavioural control, attitude, & perceived norm) with the intention of HCWs to receive LTBI treatment. The outcomes of the bivariate analysis revealed that nine factors were significantly correlated with the intention among HCWs to receive LTBI treatment. A significant link was noted between gender and intention among HCWs to receive LTBI treatment (p < 0.05). The results showed that more female HCWs (61.3%) displayed a strong intention to receive LTBI treatment than male HCWs (39.8%). A significant relationship was observed between annual household income and intention of HCWs to receive LTBI treatment (p < 0.05). Interestingly, the intention to receive LTBI treatment was significantly stronger among HCWs who earned an annual household income of ≥ RM 58,200 (62.1%) than those who earned < RM 58,200 (48.4%). The highest risk behaviour of HCWs that was significantly related to intention to receive LTBI treatment was smoking status (p < 0.05). However, more non-smokers (56.5%) had stronger intent to receive LTBI treatment than smokers (26.9%). Turning to occupational factors, workstation and job categories both displayed significant linkages with intention of HCWs to receive LTBI treatment (p < 0.05). The outcomes showed that more clinical HCWs (58.9%) had stronger intention to receive LTBI treatment when compared to non-clinical HCWs (39.4%). The HCWs who worked in high-risk workstations (59.0%) exhibited stronger intention to receive LTBI treatment than 44.2% of those working in low-risk workstations. Moving on to the IBM constructs, LTBI knowledge level, attitude, perceived behavioural control, and perceived norm displayed significant correlations with the intention of HCWs in receiving LTBI treatment (p < 0.05). The intention to receive LTBI treatment was stronger among HCWs with a high LTBI knowledge level (63.4%) than those with low LTBI knowledge (47.1%). More HCWs with favourable attitude (69.3%) had a stronger intention to receive LTBI treatment when compared to the unfavourable attitude displayed by 43.2% of the respondents. Meanwhile, 69.5% and 61.5% of the respondents exhibited strongly perceived norm and behavioural control, respectively.

**Table 2:** Factors associated with intention to receive LTBI treatment among HCWs.

Characteristics	Total	Strong intention to receive LTBI treatment	Weak intention to receive LTBI treatment	*p*-value
	(n, %)	(n, %)	(n, %)	
**Total**	**256**	**137**	**119**	
*Sociodemographic*				
**Age category (years)**				
< 35	165 (64.5)	90 (54.5)	75 (45.5)	0.656
≥ 35	91 (35.5)	47 (51.6)	44 (48.4)	
**Gender**				
Male	93 (36.3)	37 (39.8)	56 (60.2)	<0.001*
Female	163 (63.7)	100 (61.3)	63 (38.7)	
**Education level**				
Secondary level	18 (7.0)	6 (33.3)	12 (66.7)	0.075
Tertiary level	238 (93.0)	131 (55.0)	107 (45.0)	
**Annual household income category (RM)**				
< 58,200	161 (62.9)	78 (48.4)	83 (51.6)	0.034*
≥ 58,200	95 (37.1)	59 (62.1)	36 (37.9)	
**Comorbidity**				
Yes	46 (18.0)	27 (58.7)	19 (41.3)	0.437
No	210 (82.0)	110 (52.4)	100 (47.6)	
*Risk behaviour*				
**Smoking status**				
Yes	26 (10.2)	7 (26.9)	19 (73.1)	0.004*
No	230 (89.8)	130 (56.5)	100 (43.5)	
**Alcohol consumption**				
Yes	3 (1.2)	1 (33.3)	2 (66.7)	0.599
No	253 (98.8)	136 (53.8)	117 (46.2)	
*Occupational factors*				
**Job categories**				
Clinical	185 (72.3)	109 (58.9)	76 (41.1)	0.005*
Non-clinical	71 (27.7)	28 (39.4)	43 (60.6)	
**Workstation**				
High-risk	161 (62.9)	95 (59.0)	66 (41.0)	0.022*
Non-high-risk	95 (37.1)	42 (44.2)	53 (55.8)	
**History of TB closecontact**				
Yes	10 (3.9)	6 (60.0)	4 (40.0)	0.755
No	246 (96.1)	131 (53.3)	115 (46.7)	
**Level of LTBI Knowledge**				
High	101 (39.5)	64 (63.4)	37 (36.6)	0.011*
Low	155 (60.5)	73 (47.1)	82 (52.9)	
**Attitude towards LTBI treatment**				
Favourable	101 (39.5)	70 (69.3)	31 (30.7)	<0.001*
Unfavourable	155 (60.5)	67 (43.2)	88 (56.8)	
**Perceived norm towards LTBI treatment**				
Strong	95 (37.1)	66 (69.5)	29 (30.5)	<0.001*
Weak	161 (62.9)	71 (44.1)	90 (55.9)	
**Perceived behavioural control towards LTBI treatment**				
Strong	122 (47.7)	75 (61.5)	47 (38.5)	0.015*
Weak	134 (52.3)	62 (46.3)	72 (53.7)	

*Significant *p*-value < 0.05

Table 3 showed the multiple logistic regression model for predictors of good intention to receive LTBI treatment. To execute this multivariate analysis, 12 independent variables (significant at p < 0.25 for individual & interacting variables) were selected from the univariate analysis. The parsimonious model fit the data rather well due to the inclusion of relevant variables to describe the results, thus preserving the interpretability and predictability of the model. The Backward Wald approach was used to determine five statistically significant predictors, namely: LTBI knowledge, attitude, perceived norm, gender, as well as the interacting variables of perceived norm and attitude. These predictors fit the Hosmer and Lemenshow goodness of fit test (x2 = 9.783, d.f = 8, p = 0.281). The model explained between 19.3% (Cox and Snell R square) and 25.8% (Nagelkerke R squared) of the variance in intention to receive LTBI treatment. The ROC curve for the final model demonstrated a significant difference (p<0.001) with a 74% area under the curve (0.739, C.I:0.678–0.800). The value of the AUC is 0.739, suggesting that there’s a 73.9% chance that the model will correctly distinguish between those HCWs with good and poor intention to receive LTBI treatment. This relatively high AUC and its significant p-value imply that the model in this study has good predictive accuracy. As revealed by the model, the female HCWs displayed 2.03 times more likelihood to have a positive intention to receive LTBI treatment than the male HCWs (aOR: 2.033, 95% CI: 1.080–3.823). Next, those with high LTBI knowledge exerted 1.9 times more likelihood to have a good intention to receive LTBI treatment (aOR: 1.926, 95% CI: 1.093–3.397). The HCWs respondents with favourable attitude exhibited 3.7 times more likelihood of having good intent to receive LTBI treatment (aOR: 3.771, 95% CI: 1.759–8.084). Lastly, those with strongly perceived norm displayed 4.6 times higher likelihood to accept LTBI treatment (aOR: 4.593, 95% CI: 2.104–10.023). Turning to the interacting variables, both strongly perceived norm and favourable attitude of the HCWs showed less likelihood to have good intention in receiving LTBI treatment (aOR: 0.254, 95% CI: 0.078–0.827).The final predictive model derived from multiple logistic regression analysis is as follows:

Log (odds of intention to receive LTBI treatment) = -2.332 + 0.709 (female) + 0.656 (good LTBI knowledge) + 1.327 (favorable attitude) + 1.524 (good perceived norms)– 1.371 (favourable attitude*good perceived norm).

**Table 3:** Predictor for strong intention to receive LTBI treatment among HCWs.

Characteristics	Coefficient	Crude Odd Ratio (OR)	*p*-value	Coefficient	Adjusted Odd Ratio (aOR)	*p*-value
		[95% CI]			[95% CI]	
**Age category (years)**						
< 35		REF				
≥ 35	-0.116	0.890 [0.533–1.486]	0.656			
**Gender**						
Male		REF				
Female	0.876	2.402 [1.426–4.046]	<0.001*	0.709	2.033 [1.080–3.823]	0.028*
**Education level**						
Secondary level		REF				
Tertiary level	0.896	2.449 [0.889–6.741]	0.083	0.907	2.476 [0.765–8.009]	0.130
**Annual household income category (RM)**						
< 58,200		REF				
≥ 58,200	0.556	1.744 [1.040–2.925]	0.035*			
**Comorbidity**						
No		REF				
Yes	0.256	1.292 [0.677–2.466]	0.437			
**Smoking status**						
No		REF				
Yes	-1.261	0.283 [0.115–0.701]	0.006*	-0.723	0.485 [0.165–1.425]	0.188
**Alcohol consumption**						
No		REF				
Yes	-0.844	0.430 [0.039–4.804]	0.493			
**Job categories**						
Non-clinical		REF				
Clinical	0.790	2.203 [1.260–3.852]	0.006*			
**Workstation**						
Non-high-risk		REF				
High-risk	0.597	1.816 [1.088–3.032]	0.022*			
**History of TB close contact**						
No		REF				
Yes	0.275	1.317 [0.363–4.782]	0.676			
*IBM constructs*						
**LTBI Knowledge**						
Low		REF				
High	0.664	1.943 [1.163–3.246]	0.011*	0.656	1.926 [1.093–3.397]	0.023*
**Attitude**						
Unfavourable		REF			REF	
Favourable	1.087	2.966 [1.748–5.033]	<0.001*	1.327	3.771 [1.759–8.084]	<0.001*
**Perceived norm**						
Weak		REF			REF	
Strong	1.059	2.885 [1.688–4.931]	<0.001*	1.524	4.593 [2.104–10.023]	<0.001*
**Perceived behavioural**						
**control**						
Weak		REF			REF	
Strong	0.617	1.853 [1.126–3.050]	0.015*	0.350	1.418 [0.805–2.499]	0.226
*Interaction variables*						
**Attitude** * **Perceived norm**						
Weak					REF	
Strong				-1.371	0.254 [0.078–0.827]	0.023*
**Attitude with weak**						
**perceived norm**						
Weak		REF				
Strong	1.425	4.157 [1.992–8.677]	<0.001*			
**Attitude with strongly**						
**perceived norm**						
Weak		REF				
Strong	-0.064	0.938 [0.380–2.316]	0.889			
**Perceived norm with**						
**unfavourable attitude**						
Weak		REF				
Strong	1.627	5.088 [2.034–11.236]	<0.001*			
**Perceived norm with**						
**favourable attitude**						
Weak		REF				
Strong	0.094	1.099 [0.466–2.594]	0.830			

*Significant p-value at < 0.05

## Discussion

Studies concerning the intention of HCWs to receive LTBI treatment within the context of Malaysia are in scarcity. The outcomes of this study disclosed that 53.5% of the HCWs serving in the selected teaching hospital had positive intention to receive LTBI treatment. The reported figure, however, was lower than that reported in 2017 for the Eastern China context displaying 62.3% willingness to receive LTBI treatment [46]. In India, 73.5% of non-HCWs with TB close contact disclosed positive intention to receive LTBI treatment [47]. The discrepancy in intention levels with similar studies from Eastern China and India could be affected by infrastructural, cultural, and social variances. The number of HCWs in Eastern China willing to receive LTBI treatment was higher ascribable to high occupational exposure risk to contract TB. Meanwhile, a higher percentage of Indians had stronger and more positive intent to receive LTBI treatment because of their close contact with TB patients in their households. Evidently, the two studies from India and Eastern China had samples that were in close contact with TB patients or worked around them [46, 47]. Such a situation led the samples to have a high willingness to seek LTBI treatment stemming from high susceptibility to contracting TB [48].

Turning to this present study, most of the HCWs displayed weak characteristics based on the IBM model; low LTBI knowledge (60.5%), unfavourable attitude (60.5%), weakly perceived norm (62.9%), and weakly perceived behavioural control (52.3%) towards the intention to receive LTBI treatment. These outcomes reflect the limited awareness of the National LTBI program among HCWs. Despite the prolonged existence of TB, a comprehensive Global LTBI Management Framework was only recently published in 2018 by the WHO [2]. While Malaysia initiated its National TB Control Program back in 1961, comprehensive guidelines for LTBI management were released in 2021 through a directive order from the Director of the Disease Control Division, MOH [2, 18]. As the LTBI management guidelines concentrate on MOH facilities, HCWs in private sectors and teaching hospitals may receive less attention [2]. This discrepancy amplifies a wide time gap in the formulation of LTBI policies when compared to TB programs. Hence, one may say that the implementation of the LTBI program is relatively new in Malaysia and this factor might have hindered its acceptance and adherence among HCWs and the public. Limited healthcare resources may further compound the challenge of aligning with government strategies to promote LTBI treatment, particularly among emerging health concerns. While educational materials on LTBI might be scarce in Malaysia, the rapid growth of the internet may offer greater access to pertinent information. To date, HCWs can readily access up-to-date LTBI information from a wide range of online channels, such as freely available resources and social media. Besides, the digital landscape presents plenty of opportunities to gain knowledge about LTBI, thus bridging the knowledge gap. Hence, a concerted effort is imperative to harness online resources effectively so that awareness and knowledge about LTBI can be enhanced among HCWs.

Gender emerged as a significant factor in determining intent to receive LTBI treatment, given that female HCWs displayed 2.03 times more willingness to do so than male HCWs in this study. In a similar vein, male HCWs in South Korea exhibited 1.2 times higher chance of refusing LTBI treatment despite a positive diagnosis [49]. On the contrary, male HCWs in the US showed 1.9 times more likelihood to accept LTBI treatment than female HCWs [21]. Gender variance in determining one’s intention to receive LTBI treatment is attributable to numerous factors, including the impacts of individual, social, and cultural aspects on risk perception. The stronger intent displayed by female HCWs to accept LTBI treatment is ascribed to the combination of low-risk behaviour and high-risk perception [50, 51]. A proxy study on risk perception towards Coronavirus Disease 2019 (COVID-19) is applicable to explain the plausible reason why gender is linked with risk perception. For instance, a study that involved 21,000 US citizens found that females perceived higher risks of COVID-19 infection and mortality than males [52].

In this present study, female HCWs disclosed lower engagement in high-risk behaviour, such as smoking and alcohol consumption. As a result, increased health-seeking behaviour and better health literacy among females contributed to their proactive approach to healthcare [53–56]. This higher awareness encourages female HCWs to seek preventive treatment, thus in line with their intention in seeking LTBI treatment.

Another significant predictor identified in this study refers to LTBI knowledge. Respondents with high LTBI knowledge displayed 1.9 times more likelihood to accept LTBI treatment than those with low LTBI knowledge. Similarly, a survey conducted in 2013 involving the US population showed that high TB knowledge increased their willingness to undergo LTBI treatment in comparison to those with low TB knowledge [20]. Another study conducted in 2020 involving eight countries disclosed that sufficient TB knowledge served as a predictor to accept LTBI treatment among households with TB close contact [aOR 2.22, 95% CI = 1.23–3.99] [57]. Seemingly, high-level LTBI knowledge increases one’s comprehension of LTBI and promotes LTBI treatment acceptance [20]. Logically, knowledgeable HCWs about TB would have a better awareness of LTBI susceptibility given the high-risk occupational exposure [46]. Additionally, HCWs with high knowledge about the importance of LTBI treatment in hindering LTBI recurrence and other complications would have stronger intentions to accept LTBI treatment [2, 58]. Detailed information about LTBI demystifies hoaxes about the protection duration of BCG vaccination, while concurrently allowing one to weigh the benefits and risks of accepting LTBI treatment [59]. Senior HCWs were sceptical about undergoing LTBI treatment because of the reported negative effects on the elderly [49]. Given that most of the HCWs were young and free from any comorbidity, side effects from LTBI treatment were not a major concern and this fact led to a stronger intention among them to accept LTBI treatment [49, 60]. Based on the IBM, sufficient and correct knowledge about behaviour, its advantages, and its performance can intensify one’s behavioural intention. Moreover, many studies support knowledge as a crucial predictor of intention for health behaviour models [22, 58, 61].

It was found in this study that HCWs with favourable attitude displayed 2.3 times more likelihood of having stronger intention to accept LTBI treatment. Similarly, a survey executed in 2013 involving the US and Canadian public showed that those with unfavourable attitude towards LTBI treatment were more likely to refuse that treatment than those with favourable attitude [aOR 0.06, 95% CI: 0.040–0.090] [20]. Referring to the IBM, a favourable attitude contributes to predicting behavioural intention. When individuals have positive emotional responses and believe in the benefits of the behaviour, their attitude towards it becomes positive and this leads to a higher intention to engage in that behaviour [22, 58]. This aligns with the Cognitive Consistency Theory, as people strive for harmony between thoughts and actions. Positive attitude is often linked to perceived positive outcomes, which act as rewards that motivate behaviour [62, 63]. As for the HCWs in this study, perceiving LTBI treatment as preventing infection transmission, protecting patients and colleagues, as well as preserving their health elevated intention for treatment. Overall, a favourable attitude displayed a strong impact on decision-making and intention, thus making the behaviour appealing and achievable. Hence, a positive attitude towards LTBI treatment can reliably predict the intention of HCWs to accept the treatment.

Respondents with strongly perceived norm showed 2.1 times higher probability of having the intention to accept LTBI treatment. Similarly, a study conducted in 2019 among Korean HCWs reported that those with strongly perceived norm were 3.3 times more likely to have an intention to receive LTBI treatment [aOR: 3.33, 95% CI: 1.780–6.230] [64]. Turning to this present study, most of the HCWs believed that it was crucial to undergo LTBI treatment because they were encouraged by people whom they perceived as important. Simply put, the perceived norm of HCWs was shaped by the viewpoints of people important to them, depicting the subjective norm in IBM [22, 58]. For nurses and other HCWs apart from physicians, their perceived social expectation (subjective norm) substantially influenced their behaviour. This showed that societal pressure, such as a supervisor’s endorsement of clinical protocols, strongly affected their intention to follow the guidelines, even in cases of limited knowledge or negative attitude [65, 66]. In summary, perceived norm significantly predicted the intention of HCWs to accept LTBI treatment. Such huge impacts hinge on societal and cultural contexts, measuring tool quality, institutional support, as well as the way people interpret societal beliefs and behaviour. This underlines the significant role of social influence in guiding one’s intention, even when other factors such as knowledge and attitude are involved.

The study outcomes demonstrated that the interacting variables between perceived norm and attitude emerged as a significant predictor for the intention of HCWs to accept LTBI treatment. Notably, the link of strongly perceived norm with a favourable attitude among the respondents displayed a lower likelihood of having the intent to receive LTBI treatment; disclosing an enthralling dynamic. Based on the multiple logistic regression approach, both strongly perceived norm and favourable attitude functioned as significant predictors for intention to receive LTBI treatment for individual assessment but otherwise (no intent to accept treatment) for combined interaction of the two variables. This scenario is exemplified in the Suppression Effect Theory [67]. The contradicting outcomes are attributed to the items deployed for the perceived norm construct, which emphasise strongly perceived norm based on the perceptions and beliefs of cultural norm and individuals important to the respondents. Although the respondents had strongly perceived norm, they could still be adversely influenced by those who disregard LTBI treatment and non-supportive workplaces towards the policies of LTBI treatment. Such an intricate connection between favourable attitude and seemingly strongly (weakly in reality) perceived norm can lead to the intention to refuse LTBI treatment [49]. This particular complex interaction did not come to light when evaluated in isolation but was unravelled when the interaction between the two variables was taken into consideration.

This study utilised the IBM to assess factors that influenced the intention of HCWs to undergo LTBI treatment in a teaching hospital located in Malaysia. This study achieved a 100% response rate, thus ensuring representativeness. Some key strengths of this study are its grounding in the IBM framework, which is one of the established behaviour models in guiding health education programs, and its alignment with global health initiatives in *WHO End TB Strategy 2035* [2]. However, this study has several limitations, such as its specificity to one teaching hospital and its cross-sectional design that restricts causal inferences. The study recommends future research work to deploy established behavioural frameworks, such as IBM, to carry out longitudinal studies, expand across various settings, integrate qualitative methods for a holistic view, as well as focus on both clinical and behavioural aspects in enhancing temporal causal relationships. As for healthcare services, it is crucial to design tailored programs in order to address several factors, such as knowledge, attitude, and perceived norm of HCWs about LTBI treatment intention. Creating a supportive environment and leveraging HCWs as treatment advocates can foster positive norm and increase LTBI treatment uptake. Recognizing the need for broader epidemiological insights, the study highlights the importance of extending future research to include other educated demographic groups. Additionally, future studies will be recommended to explore rural and primary healthcare workers in order to generalize findings across a broader spectrum of healthcare services in Malaysia.

## Conclusion

In conclusion, the HCW respondents in this study demonstrated a predominantly good level of intention to accept LTBI treatment. The significant predictors towards intention to receive LTBI treatment among the HCWs included females, high level of LTBI knowledge, favourable attitude, strongly perceived norm, as well as the positive relationship between favourable attitude and strongly perceived norm. The reported outcomes highlight the importance of targeted health education and promotion among HCWs to enhance their level of LTBI knowledge, attitude, and perceived norm—significant predictors towards intensifying their intentions to receive LTBI treatment. Ultimately, boosting the acceptance of LTBI treatment among HCWs is crucial to decrease the incidence and mortality of TB, which is also in line with the *End TB Strategy 2035* launched by the WHO.
